# Häufigkeit interstitieller Lungenerkrankungen in der stationären Rheumatologie: Eine retrospektive Analyse stationärer Entlassungsdiagnosen von 2005 bis 2023 in Deutschland

**DOI:** 10.1007/s00393-025-01675-3

**Published:** 2025-07-11

**Authors:** Falk Schumacher, Sarah Bettina Stanzel, Maximilian Zimmermann, Daniel Majorski, Maximilian Wollsching-Strobel, Katinka Albrecht, Wolfram Windisch, Johannes Strunk, Melanie Berger

**Affiliations:** 1 Fakultät für Gesundheit/Department für Humanmedizin, Universität Witten/Herdecke, Witten, Deutschland; 2 Klinik für Rheumatologie, Krankenhaus Porz am Rhein gGmbH, Urbacher Weg 19, 51149 Köln, Deutschland; 3 Lungenklinik Merheim, Kliniken der Stadt Köln GmbH, Köln, Deutschland; 4 Programmbereich Epidemiologie und Versorgungsforschung, Deutsches Rheuma-Forschungszentrum Berlin, Berlin, Deutschland

**Keywords:** Entzündlich rheumatische Erkrankungen, Rheumatoide Arthritis, Versorgungsforschung, Kollagenose, Internationale Klassifikation der Krankheiten, Inflammatory rheumatic diseases, Rheumatoid arthritis, Health services research, Connective tissue disease, International Classification of Diseases

## Abstract

**Hintergrund:**

Die interstitielle Lungenerkrankung (ILD) bei entzündlich rheumatischen Erkrankungen (ERE) hat eine große Bedeutung in Diagnostik und Therapie. Aufgrund der oft komplexen und schweren Verläufe ist häufig eine stationäre Betreuung nötig. Bezüglich der Häufigkeit einer Lungenbeteiligung allgemein und der Entwicklung des Anteils an stationär behandelten Betroffenen in Deutschland werden neue Daten benötigt.

**Methoden:**

Es erfolgte eine retrospektive Analyse der vom statistischen Bundesamt veröffentlichten stationären Entlassungsdiagnosen nach der Internationalen Klassifikation der Krankheiten (ICD-10) aus den Jahren 2005 bis 2023 in Deutschland. Hierbei wurden 11 rheumatologische Hauptdiagnosen berücksichtigt, insbesondere rheumatoide Arthritis (M05, M06), Kollagenosen (M32–M35), ANCA-assoziierte Vaskulitiden (M30.1, M31.3, M31.7) und axiale Spondyloarthritis (M45). Die Häufigkeit zusätzlich kodierter Nebendiagnosen einer Lungenbeteiligung (J84.1, J84.8, J84.9, J99.0*, J99.1*) wurde über einen Beobachtungszeitraum von insgesamt 19 Jahren dargestellt.

**Ergebnisse:**

Pro Jahr gab es zwischen 50.540 und 64.004 stationäre Aufenthalte mit einer der untersuchten rheumatologischen Hauptdiagnosen. Die Zahl stieg von 2005 bis 2019 auf bis zu 64.000 an, brach 2020 bis 2022 ein und lag 2023 bei 54.077. Der Anteil mit einer kodierten Lungenbeteiligung stieg von 7 % (3524 in 2005) auf 17 % (9398 in 2023) an. Am häufigsten war eine Lungenbeteiligung bei Granulomatose mit Polyangiitis (46 %), systemischer Sklerose (44 %), Sjögren-Syndrom (27 %) und Sharp-Syndrom (24 %). Bei seropositiver RA hatten 8 %, bei seronegativer RA 1 % eine Lungenbeteiligung (Daten von 2023).

**Diskussion:**

Im Untersuchungszeitraum hat die Zahl stationärer Aufenthalte mit rheumatologischer Hauptdiagnose in Deutschland zugenommen – mit einem Einbruch in der der SARS-CoV-2-Pandemie. Der kontinuierlich gestiegene Anteil an Erkrankten mit einer Lungenbeteiligung weist auf den stationären interdisziplinären Versorgungsbedarf dieser wachsenden rheumatologischen Patientengruppe hin.

## Abstract (en)

**Background:**

Interstitial lung disease (ILD) in inflammatory rheumatic diseases (IRD) is of great importance in the diagnostics and treatment. Due to the often complex and severe courses, inpatient care is often necessary. New data are needed regarding the frequency of lung involvement in general and the development of the proportion of inpatients in Germany.

**Methods:**

A retrospective analysis of the inpatient diagnoses published by the Federal Statistical Office according to the International Classification of Diseases (ICD-10) from 2005 to 2023 in Germany was carried out. A total of 11 main rheumatological diagnoses were analyzed, in particular rheumatoid arthritis (RA, M05, M06), connective tissue disease (M32–M35), ANCA-associated vasculitis (M30.1, M31.3, M31.7) and axial spondylarthritis (M45). The frequency of additionally coded secondary diagnoses of lung involvement (J84.1, J84.8, J84.9, J99.0*, J99.1*) was presented over a total observational period of 19 years.

**Results:**

There were between 50,540 and 64,004 inpatient stays per year with one of the main rheumatological diagnoses examined. The number rose to as high as 64,000 from 2005 to 2019, collapsed in 2020–2022 and stood at 54,077 in 2023. The proportion with coded lung involvement rose from 7% (3524 in 2005) to 17% (9398 in 2023). Lung involvement was most common in granulomatosis with polyangiitis (46%), systemic sclerosis (44%), Sjögren’s syndrome (27%) and Sharpʼs syndrome (24%). In seropositive RA 8% and in seronegative RA 1% had lung involvement (data from 2023).

**Discussion:**

During the period under study, the number of inpatient stays with a main rheumatological diagnosis in Germany increased, with a slump during the SARS-CoV 2 pandemic. The continuously increasing proportion of patients with lung involvement indicates the inpatient interdisciplinary care needs of this growing rheumatological patient group.

## Hintergrund

Im klinischen Alltag ist die Lungenbeteiligung bei entzündlich rheumatischen Erkrankungen (ERE) ein bedeutender Faktor in der Diagnoseeinordung, Therapiesteuerung und Abschätzung der Prognose. Hierbei zeigt sich bei den Verschiedenen ERE ein heterogenes Bild hinsichtlich der Häufigkeit, der Ausprägung und der therapeutischen Relevanz einer Lungenbeteiligung [1]. Im Rahmen der jeweiligen Erkrankung kann es auch neben einer interstitiellen Lungenerkrankung (ILD) zu anderen pulmonalen Beteiligungen kommen. Relevant sind hierbei auch Erkrankungen der Atemwege, der Pleura, die Vaskulitis und pulmonale Infektionen, die durch die jeweilige Therapie begünstigt werden können [2, 3]. Die ILD stellt je nach Verlauf und diagnostischem Muster häufig einen besonders wichtigen Bereich der Lungenbeteiligung für das Management der Erkrankten dar. Diese zeigt sich im klinischen Erscheinungsbild äußerst variabel und kann in den verschiedenen Phasen der Erkrankungen auftreten. Durch den relevanten Einfluss auf die Morbidität und Mortalität spielt das Auftreten einer ILD eine bedeutende Rolle im therapeutischen Management von Erkrankten mit ERE [4–6].

Die rheumatoide Arthritis (RA) ist die ERE mit der höchsten Prävalenz in Deutschland [7]. In einer aktuellen Metaanalyse wird die Prävalenz einer ILD bei der RA mit 11 % angegeben [1]. Die Häufigkeit einer bildmorphologischen ILD in der hochauflösenden Computertomographie (HRCT) ist hierbei deutlich höher als die einer klinisch manifesten ILD [8]. Positive Rheumafaktoren bzw. anti-citrullinierte Proteinantikörper (ACPA) sind neben Rauchen und männlichem Geschlecht wichtige Risikofaktoren für die Entstehung einer ILD [9]. Die höchste Prävalenz einer ILD im Rahmen von ERE wird aktuell beim Sharp-Syndrom (56 %), der systemischen Sklerose (47 %) und den idiopathisch inflammatorischen Myopathien (IIM) (41 %) beschrieben. Eine ILD beim Sjögren-Syndrom (17 %) und beim systemischen Lupus erythematodes (6 %) ist im Vergleich deutlich geringer [1]. Bei der Spondylitis ankylosans (SpA) ist die ILD bislang nicht als häufige klinisch manifeste Organbeteiligung beschrieben [10]. In der Gruppe der Anti-Neutrophile cytoplasmatische Antikörper (ANCA)-assoziierten Vaskulitiden ist die Lungenbeteiligung zwar häufig, aber hinsichtlich ihrer anatomischen Zuordnung besonders vielseitig. In der aktuellen Literatur werden Häufigkeiten für die Granulomatose mit Polyangiitis (GPA) von 52–94 %, für die mikroskopische Polyangiitis (MPA) von 25–69 % und für die eosinophile Granulomatose mit Polyangiitis (EGPA) von 60–100 % angegeben. Eine Lungenfibrose wird am häufigsten bei der MPA beschrieben, wobei bei der GPA eher granulomatöse Veränderungen auftreten [11].

Dieses heterogene Bild einer Lungenbeteiligung bei ERE stellt eine große Herausforderung für die korrekte Darstellung in der Internationalen Klassifikation der Krankheiten(ICD)-Kodierung von stationären Fällen dar. Die Hauptdiagnose während eines stationären Aufenthaltes ist bei den rheumatologischen Diagnosen klar im System der ICD definiert. Diese wird gemäß der deutschen Kodierrichtlinien als die Diagnose definiert, die hauptsächlich für die Veranlassung des stationären Krankenhausaufenthaltes des Betroffenen ursächlich ist [12]. Bei der Nebendiagnose einer Lungenbeteiligung gibt es in dem ICD-System je nach Hauptdiagnose verschiedene Möglichkeiten der Kodierung, welche eine genaue Differenzierung einer ILD von einer anderen Form einer Lungenbeteiligung erschwert.

In einer aktuellen Arbeit auf Grundlage von Krankenkassendaten aus Deutschland wird eine Prävalenz der RA-ILD bei der seropositiven RA von 2,1–3,0 % und bei der seronegativen RA von 1,3–1,9 % beschrieben [13]. Die Häufigkeit einer ILD bei stationär-rheumatologisch behandelten Betroffenen in Deutschland ist hingegen nicht ausführlich untersucht. Aufgrund der möglichen lebensbedrohlichen Verläufe ist die stationäre Versorgung dieser Erkrankten für eine frühe Diagnostik und einen engen interdisziplinären Austausch bezüglich des therapeutischen Vorgehens oftmals nötig.

## Zielsetzung

In dieser Arbeit soll über die ICD-Kodierungen von stationären rheumatologischen Fällen ein Hinweis für die Häufigkeit einer ILD bei Erkrankten mit einer ERE in Deutschland gegeben werden. Darüber hinaus sollen diesbezüglich eine Differenzierung in den relevantesten Hauptdiagnosegruppen und eine Beobachtung des zeitlichen Verlaufes erfolgen, um Grundlagen für mögliche strukturelle Verbesserungen der Versorgung dieser Patientengruppe zu ermöglichen.

## Methoden

### Datenquelle

Es erfolgt eine retrospektive Analyse der vom statistischen Bundesamt (StBA) veröffentlichten Entlassungsdiagnosen von stationär behandelten Betroffenen aus den Jahren 2005 bis 2023 in Deutschland, welche gemeinsam mit den im StBA tätigen Datenmanagern aufbereitet wurden [14]. Durch das Krankenhausentgeltgesetz ist die Übermittlung der Leistungsdaten anhand der internationalen statistischen Klassifikation der Krankheiten (International Classification of Diseases [ICD]) für jede Einrichtung, die stationäre Betroffene behandelt, verpflichtend [15].

Erfasst werden auch die im Krankenhaus Verstorbenen, nicht jedoch teilstationär oder ambulant behandelte Erkrankte. Bei mehrfach im Jahr vollstationär Behandelten wird für jeden Krankenhausaufenthalt jeweils ein Datensatz erstellt. Erfasst wird jede vollstationäre Behandlung im Krankenhaus, unabhängig von der Zahl der dabei durchlaufenen Fachabteilungen [14].

### Definition der Diagnosen

Es wurden 11 rheumatologische Hauptdiagnosen berücksichtigt: M05: Seropositive rheumatoide Arthritis (+RA), M06: seronegative rheumatoide Arthritis (‑RA), M30.1: Eosinophile Granulomatose mit Polyangiitis (EGPA), M31.3: Granulomatose mit Polyangiitis (GPA), M31.7: Mikroskopische Polyangiitis (MPA), M32: Systemischer Lupus erythematodes (SLE), M33: Dermatomyositis-Polymyositis (DM/PM), M34: Systemische Sklerose (SSc), M35.0: Sjögren-Syndrom (SjS), M35.1: Sharp-Syndrom (Mischkollagenose), M45.0: Spondylitis ankylosans (SpA).

Für das Vorhandensein einer ILD wurden folgende Nebendiagnosen berücksichtigt: J84.1: Sonstige interstitielle Lungenkrankheiten mit Fibrose, J84.8: Sonstige näher bezeichnete interstitielle Lungenkrankheiten, J84.9: Interstitielle Lungenkrankheit, nicht näher bezeichnet, J99.0*: Lungenkrankheit bei seropositiver chronischer Polyarthritis, J99.1*: Krankheiten der Atemwege bei sonstigen diffusen Bindegewebskrankheiten. Hierbei ist zu beachten, dass alle Nebendiagnosen außer J99.1* gezielt auf eine ILD zu beziehen sind. J99.1* kann auch bei anderen Lungenveränderungen im Rahmen einer ERE kodiert werden. Daher erfolgte ergänzend eine Auswertung ohne J99.1*. Für andere Formen der Lungenbeteiligung wie einem Pleuraerguss oder einer subglottischen Stenose existieren alternative Verschlüsselungsformen, welche nicht in die Analyse integriert wurden.

### Auswertungen

Die Anzahl an Krankenhausaufenthalten mit rheumatologischer Hauptdiagnose und mit Nebendiagnose einer ILD wird für jedes Jahr und für die verschiedenen Diagnosegruppen dargestellt.

Die statistische Analyse erfolgte auf Grundlage der vom statistischen Bundesamt zur Verfügung gestellten Datensätze der Jahre 2005 bis 2023. Die Daten wurden durch Mittelwerte und Streuungen (Standardabweichung) beschrieben. Die statistischen Analysen wurden mit Microsoft Excel Version 2307 (Microsoft, Redmond, Washington, USA) durchgeführt.

## Ergebnisse

Die Analyse umfasst insgesamt 10,8 Mio. stationäre Aufenthalte mit einer der 11 berücksichtigten rheumatologischen Hauptdiagnosen über einen Beobachtungszeitraum von 19 Jahren. Bei 142.189 Aufenthalten (13 %) wurde eine Lungenbeteiligung als Nebendiagnose dokumentiert. Im Mittel gab es pro Jahr 57.054 (SD 4821) Aufenthalte mit einer rheumatologischen Hauptdiagnose, und davon hatten 2484 (SD 2040) eine Lungenbeteiligung.

### Anzahl an Krankenhausaufenthalten mit rheumatologischer Entlassungsdiagnose von 2005 bis 2023

**Abb. 1:**
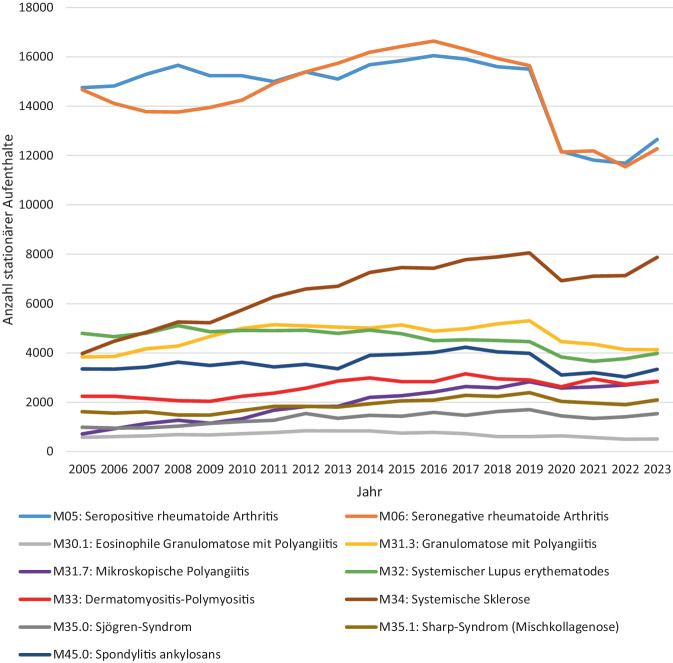
Anzahl stationärer Aufenthalte mit rheumatologischer Hauptdiagnose von 2005 bis 2023

**Abb. 2:**
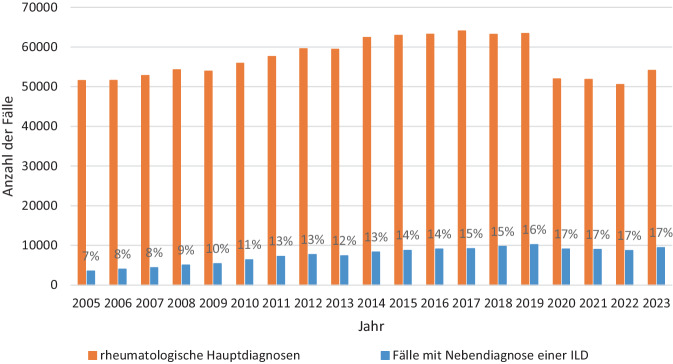
Summe der rheumatologischen Fälle und Nebendiagnosen einer Lungenbeteiligung im zeitlichen Verlauf. Die Balken veranschaulichen die absoluten Zahlen, die Prozentzahlen zeigen jeweils den Anteil der Fälle mit Lungenbeteiligung in dem jeweiligen Jahr. *ILD* interstitielle Lungenerkrankung

Mit ca. 30.000 stationären Aufenthalten pro Jahr stellt die RA etwa die Hälfte aller stationären Diagnosen mit gleichen Anteilen seropositiver und seronegativer RA dar. Diese wird gefolgt von der SSc (≈ 6500/Jahr), GPA (≈ 4700/Jahr) und SLE (≈ 4600/Jahr) (Abb. 1). Von 2005 bis 2019 stieg die Anzahl stationärer Aufenthalte bei rheumatologischer Entlassungsdiagnose insgesamt von 51.532 auf 63.383 (+23 %). Am stärksten zeigt sich dieser Anstieg bei der SSc von 3975 auf 8056 (+103 %). In den Jahren 2020 und 2021 waren die stationären Aufenthalte in allen Diagnosegruppen rückläufig (−18 %). In 2023 stiegen die Zahlen wieder leicht an, wobei in den meisten Diagnosen nicht das Ausgangsniveau von 2019 erreicht wurde (Abb. 2).

### Anteil der Fälle mit Lungenbeteiligung als Nebendiagnose (2005 bis 2023)

Die Abb. 2 zeigt den Anteil stationärer Aufenthalte für die Summe aller ausgewerteten rheumatologischen Hauptdiagnosen pro Jahr. Bei der Betrachtung der Aufenthalte mit einer Lungenbeteiligung als Nebendiagnose zeigt sich ein ähnlicher zeitlicher Trend mit einer Zunahme der absoluten Fallzahlen mit einer Lungenbeteiligung von 2005 (*n* = 3524) bis 2023 (*n* = 9398). Der Anteil an kodierten ILD-Nebendiagnosen an der Gesamtheit der beobachteten stationären Fälle hat im Beobachtungszeitraum von 7 % auf 17 % zugenommen. Der prozentuale Anteil der der Betroffenen mit einer Nebendiagnose einer ILD blieb seit Beginn der COVID-19-Pandemie bis 2023 konstant.

### Anteil mit Lungenbeteiligung bei verschiedenen Diagnosen

**Abb. 3:**
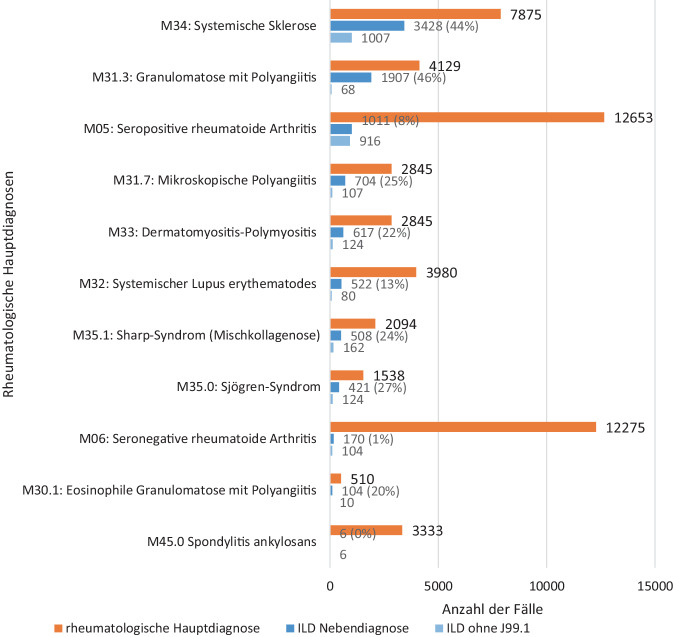
Häufigkeit der verschiedenen rheumatologischen Hauptdiagnosen und der Anteil an Fällen mit Lungenbeteiligung für das Jahr 2023 mit und ohne J99.1*. Die Balken veranschaulichen die absoluten Zahlen, die Prozentzahlen zeigen jeweils den Anteil der Fälle mit Lungenbeteiligung bezogen auf die Hauptdiagnose. *ILD* interstitielle Lungenerkrankung

Am häufigsten wurde eine Lungenbeteiligung bei Granulomatose mit Polyangiitis (46 %), systemischer Sklerose (44 %), Sjögren-Syndrom (27 %) und Sharp-Syndrom (24 %) kodiert (Abb. 3). Unter den ANCA-assoziierten Vaskulitiden zeigt sich in der größten Gruppe der GPA sowohl der größte absolute als auch der größte relative Anteil an Erkrankten mit einer Lungenbeteiligung (*n* = 1907, 46 %). Bei den Kollagenosen wurden höhere Anteile an ILD-bezogenen ICDs als bei RA dokumentiert, wobei die SSc als häufigste Kollagenose auch am häufigsten eine Lungenbeteiligung zeigt. Bei seropositiver RA hatten 8 %, bei seronegativer RA 1 % eine Lungenbeteiligung. Bei Hauptdiagnose einer SpA wurden nahezu keine Fälle einer ILD kodiert.

### Anteil mit ILD-spezifischen ICD-10-Codes

Wenn von der Auswertung exemplarisch für das Jahr 2023 die Nebendiagnose J99.1* exkludiert wird, zeigen sich v. a. bei den Kollagenosen und Vaskulitiden, weniger aber bei der RA deutlich reduzierte Anteile einer ILD (Abb. 3).

**Abb. 4:**
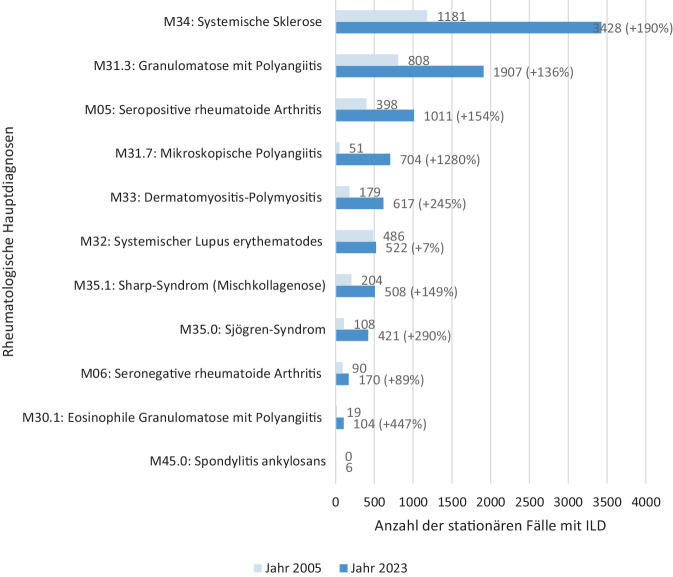
Häufigkeit der Lungenbeteiligung in den Jahren 2005 und 2023. (Die Balken veranschaulichen die absoluten Zahlen, die Prozentzahlen zeigen jeweils die prozentuale Steigerung im Jahr 2023, *ILD* interstitielle Lungenerkrankung)

**Abb. 5:**
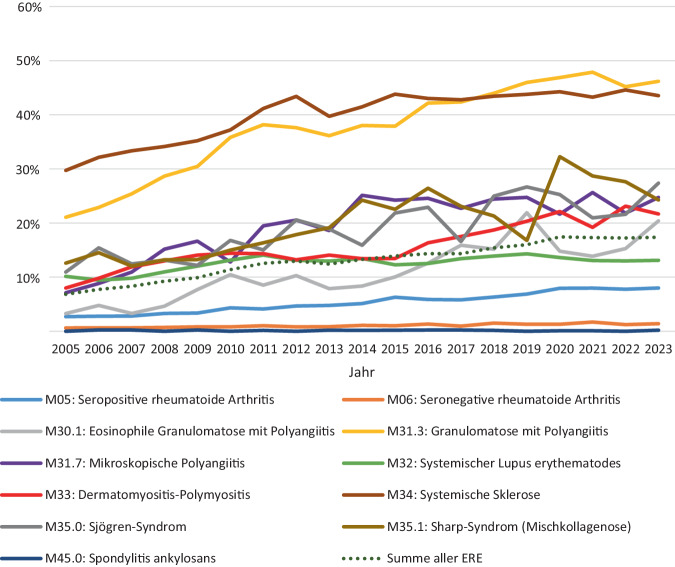
Häufigkeit der Lungenbeteiligung im zeitlichen Verlauf in Bezug auf die verschiedenen Hauptdiagnosen. *ERE* entzündlich rheumatische Erkrankungen

Die Abb. 4 verdeutlicht den Vergleich der Anteile an ILD-Fällen in den einzelnen Hauptdiagnosegruppen bezogen auf das Jahr 2005 und 2023. Bei der Betrachtung der absoluten Zahlen der behandelten Erkrankten mit Lungenbeteiligung zeigte sich, dass die systemische Sklerose bezogen auf das Jahr 2023 mit 3428 Fällen die Hauptdiagnose mit dem häufigsten Auftreten einer ILD ist, gefolgt von GPA (1907 Fälle) und seropositiver RA (1011 Fälle). Im Vergleich von 2005 und 2023 zeigen sich bei MPA, EGPA, SjS und DM/PM die größten relativen Steigerungen (Abb. 4). Die Häufigkeit der Lungenbeteiligung nimmt im Untersuchungszeitraum nahezu in allen verschiedenen Hauptdiagnosen zu (Abb. 5).

## Diskussion

In der vorliegenden Studie wird deutlich, dass über den gesamten Beobachtungszeitraum mit 13 % bei einem relevanten Anteil der stationär behandelten Betroffenen mit einer rheumatologischen Hauptdiagnose auch eine Lungenbeteiligung kodiert wurde. Bei der hohen Diversität der Grunderkrankungen und der jeweils verschiedenen möglichen Organbeteiligungen stellt diese Patientengruppe einen wichtigen Teil des therapeutischen Agierens in der stationären Rheumatologie dar. Im Untersuchungszeitraum dieser Studie zeigt sich eine maßgebliche Steigerung des Anteils an stationären rheumatologischen Fällen mit einer kodierten Nebendiagnose einer ILD. Gründe hierfür könnten eine bessere Frühdiagnostik oder die Zunahme von älteren erkrankten Personen sein. Es wäre somit relevant, eine zunehmend differenzierte Betrachtung dieser Patientengruppe zu forcieren, um die Versorgung auch in Zukunft zu optimieren.

Die Prävalenz von ERE in Deutschland ist im Zeitraum von 2014 bis 2022 gestiegen [7, 16], was die Zunahme der Gesamtheit stationärer Fälle in der vorliegenden Studie erklären kann.

Bei der differenzierten Betrachtung der verschiedenen Diagnosegruppen zeigt sich in einzelnen Gruppen der Kollagenosen und Vaskulitiden eine besonders auffällige Steigerung der Fallzahlen wie bei der SSc, DM/PM, SjS und MPA. Neben der Zunahme der Prävalenz spielen hierbei möglicherweise auch der Evidenzzuwachs und eine umfassendere frühere Diagnosestellung eine wichtige Rolle. Bei anderen Erkrankungen wie dem SLE ist eher ein Rückgang der stationären Fallzahlen insgesamt zu verzeichnen, was gegebenenfalls auch auf verbesserte ambulante Versorgungsstrukturen zurückzuführen ist. Bei der Betrachtung der Gesamtzahlen der stationären Fälle in den verschiedenen Diagnosen zeigen unsere Daten, dass die SSc in Relation zur RA hinsichtlich der Anzahl der Fälle deutlich seltener stationär behandelt wird. Eine frühere stationäre Behandlung könnte hier ggf. die Diagnostik zur Erkennung einer ILD beschleunigen und auch eine schnellere Therapieeinleitung mit den aktuell zur Verfügung stehenden Substanzen ermöglichen.

Bezüglich der COVID-19-Pandemie war in der gesamten stationären Rheumatologie ein Rückgang der Fallzahlen zu verzeichnen [17]. Dies konnte in unserer Auswertung reproduziert werden. Wie bereits in anderen Analysen gezeigt, wurden in dieser Zeit nicht unbedingt mehr Betroffene mit Organbeteiligungen in der stationären Rheumatologie versorgt [17]. Dies lässt sich durch die in dieser Arbeit gezeigten recht konstanten Anteile der Lungenbeteiligung in den Jahren 2019 bis 2020 bestätigen.

Die ICD-bezogenen Daten unserer Studie belegen den höheren Anteil einer ILD bei seropositiver RA. Der Anteil der stationären ILD-Diagnosen bei RA-Erkrankten liegt im Referenzjahr 2023 mit 5 % über den Zahlen von 1,7–2,2 % pro Jahr, welche in ambulanten Analysen erhoben wurden [13]. Dies könnte einen Hinweis darauf geben, dass der Anteil an behandelten RA-Fällen mit relevanten Organbeteiligungen wie einer RA-ILD im stationären Bereich höher ist als in der ambulanten Versorgung. Der radiologische Nachweis von interstitiellen Lungenveränderungen bei der SpA wird in der Literatur mit unterschiedlichen Zahlen (7–30 %) beschrieben [18, 19]. Die eher geringe klinische Relevanz zeigt sich auch in den Daten unserer Studie. Bezüglich der ANCA-assoziierten Vaskulitiden ist von einer sehr unscharfen Differenzierung der verschiedenen anatomischen Manifestationen einer Lungenbeteiligung durch die Kodierung von Nebendiagnosen auszugehen. Am häufigsten wurde eine Lungenbeteiligung bei der GPA dokumentiert, wobei durch die hier mit ICD-Codes beschriebenen Fälle nicht durchweg von einer klassischen ILD auszugehen ist. In dieser Gruppe ist davon auszugehen, dass ein Großteil der Betroffenen im HRCT granulomatöse Veränderungen und keine ILD zeigt. Eine differenzierte Betrachtung dieser Umstände ist auf Grundlage der ICD-Codes leider nicht möglich. In der Gruppe der Kollagenosen sind sowohl bei SjS als auch bei SLE in unserer untersuchten Gruppe höhere Anteile einer ILD als in der Literatur zu verzeichnen [1]. Dies könnte durch die Selektion von Betroffenen mit prognoserelevanter Organbeteiligung im stationären Setting im Vergleich zu der Gesamtheit der Erkrankten mit einer ERE zu erklären sein. Der hohe Anteil stationärer Fälle mit einer SSc und einer kodierten ILD steht im Einklang mit den Zahlen der Literatur [1]. Durch die hohe klinische und prognostische Relevanz der ILD bei SSc ist es wichtig, diese Betroffenen auch stationär zu versorgen. Beim Sharp-Syndrom und der DM/PM lassen sich in der stationären Versorgung geringere Anteile einer Lungenbeteiligung, als in der Literatur angegeben, verzeichnen [1]. Die mögliche Ursache einer zu geringen Fokussierung auf diese Organmanifestation könnte auf ein Verbesserungspotenzial der Diagnostik in dieser Gruppe hinweisen. Je nach diagnostischer Konstellation ist die prognostische Relevanz vor allen Dingen in der Gruppe der idiopathischen inflammatorischen Myositiden (IIM) von besonderer Bedeutung [20].

Die Zunahme der Anteile einer ILD unterscheidet sich in den einzelnen Diagnosegruppen teils erheblich. So zeigen sich bei Erkrankungen wie dem SLE oder der seronegativen RA, bei denen die klinisch manifeste ILD in der Literatur als selten angegeben wird [6], auch die geringsten Anteile der Steigerung. Bei den ANCA-assoziierten Vaskulitiden, bei denen das Bild der Lungenbeteiligung besonders heterogen ist, zeigt sich eine deutlich größere Steigerung der Anteile. Dies könnte sowohl durch eine häufige Kodierung als auch durch eine verbesserte Evidenz und Aufklärung in diesem Bereich zu begründen sein. Die weiteren Kollagenosen zeigen alle relevante Anteile an gesteigerten ILD-Fällen, was die Bedeutung dieser Diagnosegruppe für die stationäre Rheumatologie besonders deutlich macht. Neben den größeren Gruppen wie der SSc und der GPA ist auch mit einer zunehmenden Relevanz der anderen Gruppen wie des SjS und der IIM zu rechnen.

Die größte Limitation dieser Studie ist die Unschärfe der ICD-Kodierung hinsichtlich einer ILD. Der unspezifische ICD-10-Code J99.1* Krankheiten der Atemwege bei sonstigen diffusen Bindegewebskrankheiten stellt bei ERE die am häufigsten kodierte lungenbezogene Nebendiagnose dar. Die exemplarische Analyse unserer Daten ohne diesen Code führt in den meisten Diagnosegruppen zu deutlich geringen Fallzahlen. Erfahrungen aus dem Kodierverhalten an unseren Kliniken zeigen aber, dass auch dieser unspezifische ICD-Code relativ häufig bei ILD dokumentiert wird, sodass ein Ausblenden zu einer deutlichen Unterschätzung führt, die Berücksichtigung eine Überschätzung der ILD-Prävalenz mit sich bringt, da hier auch Fälle einer anderen Lungenbeteiligung als einer ILD einbezogen sein können.

Eine Stärke dieser Untersuchung ist der lange Beobachtungszeitraum mit gleicher Datenquelle, sodass Entwicklungen über die Jahre gezeigt werden konnten. Der ausgeglichene Anteil an seropositiver und seronegativer RA belegt die höhere Kodierqualität im stationären Bereich im Vergleich zu ambulanten Daten, in denen die unspezifischen Diagnosen viel häufiger verwendet werden [13].

Für eine differenzierte und effektive Diagnostik und Therapie dieser Patientengruppe ist die enge interdisziplinäre Zusammenarbeit von Rheumatologen, Pneumologen, Radiologen, Pathologen und gegebenenfalls anderen Fachbereichen leitliniengerecht im Rahmen einer multidisziplinären Konferenz/ILD-Board von besonderer Bedeutung [10]. Dies sollte bei der Bedarfsplanung für die Versorgung dieser wachsenden Gruppe berücksichtigt werden.

## Fazit für die Praxis

- In der Rheumatologie steigt der stationäre Versorgungsbedarf durch eine wachsende Zahl an Betroffenen mit einer ILD bzw. anderen Lungenbeteiligung. Auch die Pneumologie sieht sich zunehmend mit rheumatologischen Diagnosen in der stationären Versorgung von interstitiellen Lungenerkrankungen konfrontiert.
- Diese Patientengruppe erfordert eine komplexe rheumatologische sowie interdisziplinäre Diagnostik und Therapie, sodass stationäre Strukturen für die Erst- und Folgeversorgungen der oft progredienten Verläufe dieser Erkrankungen vorgehalten werden müssen.

## Data Availability

Die erhobenen Datensätze können auf begründete Anfrage in anonymisierter Form beim korrespondierenden Autor angefordert werden.
